# Time-course effects of exercise intervention on executive function in adolescents with obesity

**DOI:** 10.3389/fpsyg.2024.1346896

**Published:** 2024-09-24

**Authors:** Lingling Hu, Qiqi Shen, Hengchan Yin, Lei Cui

**Affiliations:** ^1^College of P.E. and Sports, Beijing Normal University, Beijing, China; ^2^Student Affairs Office, China Conservatory of Music, Beijing, China

**Keywords:** obese adolescents, executive functions, exercise, inhibition, working memory, cognitive flexibility

## Abstract

**Objective:**

This study was to investigate the developmental characteristics of executive function (EF) in obese adolescents and the time-course effects of a 14-week exercise intervention combining aerobic exercise and resistance training on EF in this population.

**Methods:**

The experimental group of 28 obese junior high school students participated in the exercise intervention combining aerobic exercise and resistance training, while the control group of 24 healthy weight junior high school students engaged in the regular recess exercise. EF, including inhibition, working memory, and cognitive flexibility, was assessed 1 week prior to the exercise intervention and at 12 and 14 weeks post-intervention. Changes in EF sub-functions in both groups at different time points during the exercise intervention were analyzed.

**Results:**

The findings revealed that obese junior high school students exhibited lower levels of inhibition (*p* = 0.003, Cohen’s *d* = 0.848) and cognitive flexibility (*p* = 0.013, Cohen’s *d* = 0.706) compared to their healthy weight peers. The exercise intervention combining aerobic exercise and resistance training led to significant improvements in EF among obese junior high school students, with inhibition (*p* < 0.01, Cohen’s *d* = 0.713; *p* = 0.003, Cohen’s *d* = 0.683) and cognitive flexibility (*p* = 0.001, Cohen’s *d* = 0.797; *p* < 0.01, Cohen’s *d* = 0.890) showing significant improvement at 12 and 14 weeks post-intervention, and working memory demonstrating significant improvement at 14 weeks (*p* = 0.004, Cohen’s *d* = 0.710). No significant differences were observed in EF over time in healthy weight junior high school students.

**Conclusion:**

Obese adolescents had impaired EF, as evidenced by low levels of the inhibition and cognitive flexibility compared to healthy weight adolescents. The exercise intervention combining aerobic exercise and resistance training had a positive effect on EF of obese adolescents. The time-course effects of the intervention on improvements in inhibition, working memory, and cognitive flexibility varied with intervention duration in obese adolescents, with significant changes in inhibition and cognitive flexibility observed at 12 weeks and significant changes in working memory at 14 weeks.

## 1 Introduction

Globally, there is a persistent public health concern regarding the high prevalence of overweight and obesity (Douglas et al., 2019; Taghizadeh and Farhangi, 2020; Hestbaek et al., 2021; Karampatsou et al., 2021; Clarke et al., 2022). Adolescents who are obese face adverse effects on their physical fitness and are at an elevated risk of developing chronic conditions such as diabetes and hypertension (Disse and Zimmer, 2014; Gao et al., 2022). Additionally, obesity has been found to have a negative impact on the cognitive function of adolescents (Smith et al., 2011; Bocarsly et al., 2015; Wang et al., 2016; Meo et al., 2019).

One crucial aspect of cognitive function is executive function (EF), which encompasses higher-level cognitive processes that regulate and manage various fundamental cognitive functions during the execution of complex cognitive tasks. EF comprises three sub-functions: inhibition, working memory and cognitive flexibility (Friedman et al., 2006; Diamond, 2012; Chen et al., 2019). Research indicated that obese adolescents exhibit poorer inhibition in EF compared to their healthy weight peers (Yi et al., 2015; Kittel et al., 2017; Mamrot and Hanć, 2019). Therefore, it is imperative to investigate effective strategies to enhance the EF of obese adolescents.

Moreover, prior research has indicated that exercise intervention can enhance physical fitness, motor proficiency, and academic achievement in adolescents with and without disabilities (Davis et al., 2011; Battaglia et al., 2019). These studies have also shown that exercise intervention can enhance EF in both typically developing adolescents and those disabilities who exhibit deficits in EF (Davis et al., 2011; O'Malley, 2011; Audiffren and André, 2019; Lind et al., 2019; Mehren et al., 2019; Schwarck et al., 2019). However, there was a lack of research on exercise intervention targeting EF in obese adolescents, and the progression of changes over time remained unclear. Therefore, this study aimed to investigate the developmental characteristics of EF in obese junior high school students. By focusing on the characteristics of EF, the study implemented a 14-week exercise intervention combining aerobic exercise and resistance training. The exercise intervention was designed based on three key pathways for enhancing EF through exercise: action characteristics, exercise intensity, and exercise scenarios (Pan et al., 2016). The aim of the study was to investigate the effects of exercise intervention on the EF of obese junior high school students, as well as the time-course effects, in order to develop an efficient exercise intervention regimen for enhancing the EF of obese adolescents. The objective of the research was to establish a theoretical foundation and practical guidance for utilizing exercise to enhance the physical and mental well-being of this population. The study hypothesized that obese adolescents exhibit lower EF levels compared to their healthy weight peers, and that exercise intervention can enhance the different sub-functions of EF in obese adolescents, with the effectiveness of improvement varying over time.

## 2 Materials and methods

### 2.1 Study design

A hybrid experimental design involving 2 groups (experimental group, control group) × 3 time points (pretest, 12 weeks, 14 weeks) was implemented in this study (Pan et al., 2016).

The experimental group consisted of obese junior high school students who performed an exercise intervention combining aerobic exercise and resistance training. The control group consisted of junior high school students of healthy weight who performed regular recess exercises.

The exercise intervention was administered by professional physical education instructors. Several prior research studies have demonstrated that a 12-week exercise intervention can enhance EF to different extents (Liu, 2017; Meng, 2021; He, 2022). This study aimed to investigate whether a longer duration of exercise intervention can lead to greater improvements in EF. To achieve this, the length of the exercise intervention was extended, and assessments of EF were conducted at both the 12-week and 14-week marks of the intervention period.

### 2.2 Participants

*A priori* sample size estimation was conducted using G*Power version 3.1.9.7 (Kang, 2021), with an effect size f of 0.25, *α* as 0.05, group as 2, number of measures as 2, the sample size was 28.

The obese junior high school students included in this study were selected based on specific criteria. These criteria were: (1) Body Mass Index (BMI): BMI = weight (kg)/height (m)^2^. BMI screening according to WS/T 586–2018 Screening for overweight and obesity among school-age children and adolescents (National Health and Family Planning Commission, 2018), that is, males were eligible for inclusion if they had a BMI greater than 25.2 kg/m^2^ at age 13 years and 25.7 kg/m^2^ at age 13.5 years, and females were eligible for inclusion if they had a BMI greater than 25.0 kg/m^2^ at age 13 years and 25.6 kg/m^2^ at age 13.5 years. (2) Body Fat Ratio (BFR): BFR was measured by Bioelectrical Impedance Analysis (BIA) using a Body Composition Analyzer (Brand: TANITA, Model: MC-980MA), and according to WHO guidelines (WHO Expert Committee, 1995; Chusyd et al., 2016; Tomassoni et al., 2020; Iłowiecka et al., 2021), a BFR greater than 25% for males and 35% for females was considered eligible for inclusion. (3) All participants were aged between 13 and 14 years, of normal intelligence, without a history of psychiatric or genetic disorders. They willingly volunteered for the study, provided written informed consent, and their parents were informed and consented to their participation.

In this study, cluster sampling methodology was employed to select participants. Specifically, 28 obese junior high school students were identified from a pool of 170 students, resulting in a detection rate of 16.5%. Additionally, 24 junior high school students with healthy BMI and BFR were chosen randomly to serve as the control group. The group of the experiment was double-blind, ensuring that both the subjects and the individuals administering the exercise intervention were unaware of the group assignments.

The study was carried out following the guidelines outlined in the Declaration of Helsinki, received approval from the Ethics Committee, adhered to the Standards of Ethics in Sport and Exercise Science Research, and was undertaken in partnership with the Laboratory School. The study protocol was registered with the Chinese Clinical Trial Registry at https://www.chictr.org.cn/.

### 2.3 Materials

The EF was assessed using the E-prime 2.0 system on a computer (Chen et al., 2011a,b). Participants were instructed to provide prompt and precise responses in each task, emphasizing the attainment of proficiency before progressing to the formal assessment. EF evaluations were conducted at three specific intervals: 1 week pre-intervention, 12 weeks post-intervention, and 14 weeks post-intervention. The collection of EF test data was organized by members of the research team.

**Figure 1:**
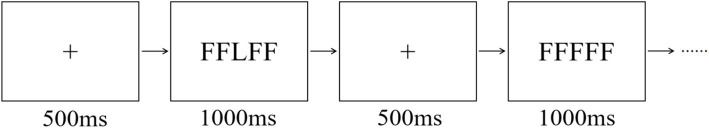
Flanker task.

In the Flanker task, participants were presented with two types of conditions: consistent conditions, where all letters on the screen are the same (e.g., “FFFFF”), and inconsistent conditions, where the middle letter was different from the others (e.g., “FFLFF”), as shown in Figure 1. Each condition was displayed for 1,000 ms with an intermediate interval of 500 ms “+.” The conditions were randomly presented with equal probability during the test, which includes 12 practice trials followed by 48 formal judgments. Test scores were calculated as the mean response time (RT) for inconsistent conditions minus that for consistent conditions, with a smaller difference indicating better inhibition performance.

**Figure 2:**
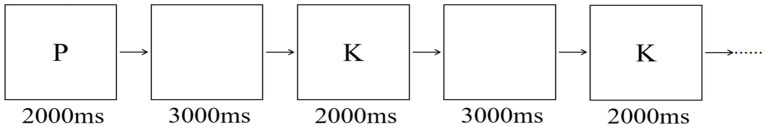
1-back task.

In the 1-back task, participants were exposed to a series of 26 English letters displayed on a screen. Each letter was shown for a duration of 2,000 ms, with a 3,000 ms interval between consecutive letters. The task required subjects to determine if the current letter matched the preceding one and respond accordingly using a designated key, as illustrated in Figure 2. Prior to the main data collection phase, participants underwent 12 practice trials. The formal task comprised 25 judgment trials. Performance on the task was assessed based on the average RT, with shorter times indicating better working memory capacity.

**Figure 3:**
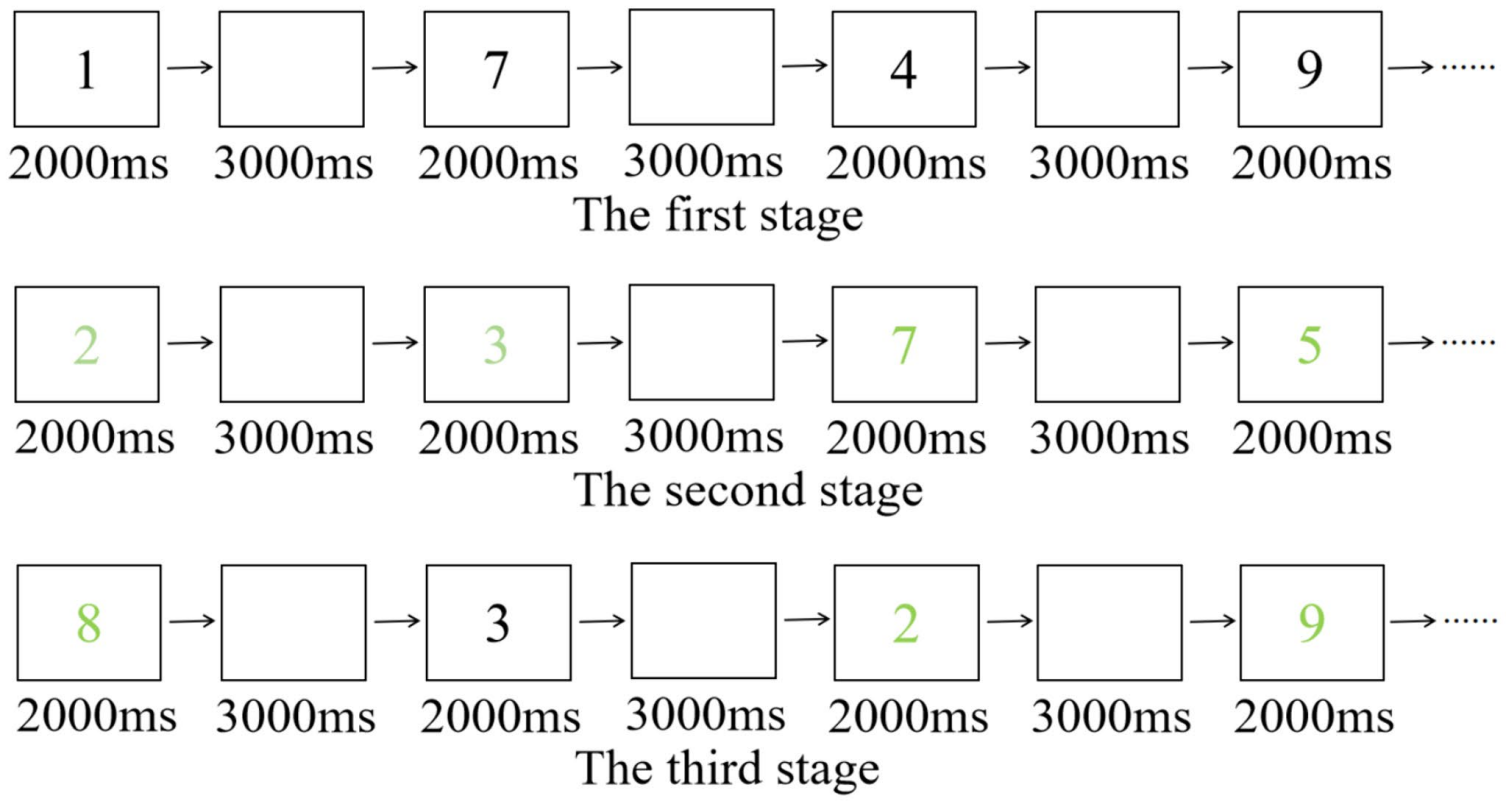
More-odd shifting task.

In the More-odd shifting task, participants were exposed to numerical stimuli, with one number displayed on each screen. Each number was shown for 2,000 ms, with a 3,000 ms interval between different digits. The test comprised three distinct stages. The initial stage involved the presentation of black numbers to assess the relationship between the number and the magnitude of 5. Subsequently, green numbers were displayed in the second stage to evaluate the parity of the numbers (odd or even). In the final stage, a mix of green and black numbers were randomly presented, with participants required to judge magnitude when black numbers appeared and parity when green numbers were shown, as illustrated in Figure 3. The first and second stages each included 8 practice trials and 16 formal judgments, while the third stage encompassed 16 practice trials and 32 formal judgments. Cognitive flexibility was evaluated by measuring the difference in RT, calculated as the average RT of the third stage minus the average RT of the first and second stages. Smaller differences indicated better cognitive flexibility.

### 2.4 Procedure

Exercise in the experimental and control groups took place at the same time. The experimental group participated in a 40-min exercise intervention combining aerobic exercise and resistance training, while the control group participated in a 40-min regular recess exercises. Both groups engaged in exercise activities during the 50-min morning school recess period. The exercise intervention was centrally administered from September to December.

The multi-path theory, as posited by our research team in the previous period, suggested that the beneficial impact of exercise intervention on EF development can be realized through three pathways (Pan et al., 2016): action characteristics, exercise intensity and exercise scenarios. Action characteristics refer to the technical characteristics of the exercise program, such as the complexity, novelty and relevance of the actions involved. Actions that were intricate, innovative, and purposeful necessitate continual adjustment and adaptation to external stimuli, thereby engaging EF in the brain. This engagement was further enhanced through repetitive training. Exercise intensity encompassed varying levels of exertion, including low, moderate, and high intensity. Research indicated that different intensities of exercise both can enhance EF, with moderate intensity exercise demonstrating the most favorable outcomes (Chen et al., 2014). Exercise scenarios referred to the instructional and learning environment in which individuals engage in physical activity. Movement learning was most effective when situated within a specific context, and the sub-components of EF can be strengthened through the implementation of cooperative, multi-memory point, multi-tasking, and problem-based scenarios.

Based on the above, in the development of an exercise intervention for obese junior high school students, in terms of action characteristics, the difficulty of the actions was gradually increased according to the subjects’ mastery of each motor skill, from single actions to combined actions, from simple to complex actions, and from familiar to unfamiliar actions. In terms of exercise intensity, a subset of participants wore Polar heart rate monitors during each exercise intervention, with average heart rates recorded at specific intervals to ensure moderate intensity levels were maintained. Furthermore, exercise scenarios were tailored to include cooperative, multi-memory point, multi-tasking, and problematic challenges aligned with participants’ preferences, gradually escalating cognitive demands to sustain engagement and enhance EF.

The 14-week exercise intervention program consisted of the following components: (1) Exercise frequency: participants engaged in physical activity 4 times per week. (2) Exercise intensity: participants exercised at a moderate intensity, calculated as (220 minus age) multiplied by 60–69%. (3) Duration of each exercise session: 40 min per session, comprising 5 min of warm-up, 20 min of aerobic exercise, 10 min of resistance training, and 5 min of relaxation exercises. (4) Type of exercise: the intervention included a combination of aerobic exercise and resistance training, with aerobic exercise being the primary focus and resistance training serving as a supplementary component. Aerobic activities included pattern running, aerobic games, and rope ladder training, while resistance training focused on exercises utilizing one’s own body weight to target strength development in the upper limbs, lower limbs, and core muscles.

### 2.5 Data analysis

SPSS 25.0 (SPSS Inc., Chicago, IL, United States) was used to process and analyze the data, the Independent-samples T test was used to developmentally characterize the EF, BMI and BFR of obese and healthy weight junior high school students, and the repeated-measures ANOVA was used to analyze the separate and interactive effects of two factors of group and time, with statistical analysis significant at *p* ≤ 0.05. Calculations of “Cohen’s effect size” and “post-hoc power analysis” using G*Power version 3.1.9.7 (Kang, 2021), considering *α* of 0.05, two groups, and three measurement points.

## 3 Results

### 3.1 Monitoring of exercise intensity

**Figure 4:**
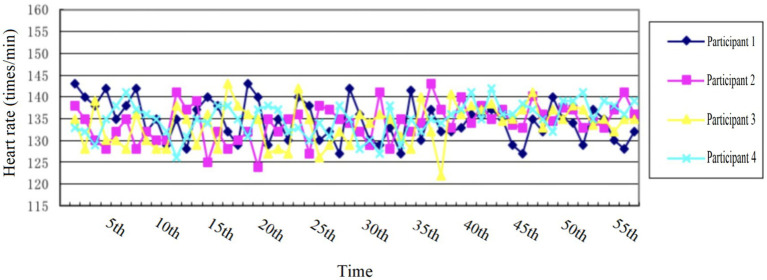
The monitoring figure of the individual average heart rate per activity of 4 obese junior high school students in the experimental group.

**Figure 5:**
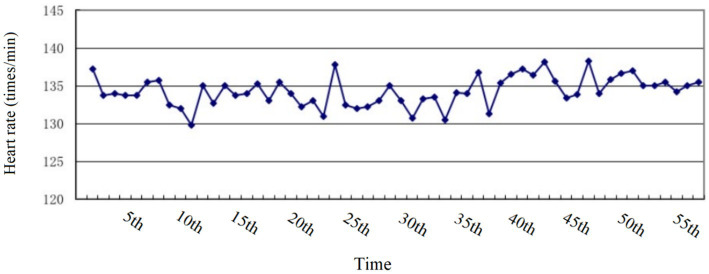
The monitoring figure of the overall average heart rate per activity of 4 obese junior high school students in the experimental group.

In order to accurately control the exercise intensity, 4 obese junior high school students were randomly selected in this study to wear Polar meter to monitor heart rate. Specific methods: 2 male and 2 female students were randomly selected, and 5 heart rate measurements were taken during each exercise intervention, namely: during the quiet time before the intervention, the 10th minute, the 20th minute, the 30th minute, and the 40th minute, and the exercise intensity was monitored according to the average heart rate. As shown in Figure 4, the average heart rate of the 4 randomly selected obese junior high school students in the experimental group during the exercise intervention was 134 beats/min, which reached about 65% of their maximum heart rate; as shown in Figure 5, the overall average heart rate of the 4 randomly selected obese junior high school students during each intervention ranged from 129 to 137 beats/min, which reached the expected moderate intensity of the exercise intervention.

### 3.2 Developmental characteristics of EF in obese junior high school students

To investigate the developmental characteristics of EF in obese junior high school students, pretest data on EF performanced in obese and healthy weight junior high school students were analyzed using Independent-samples *T-*tests. The results revealed that obese junior high school students had longer average RT for EF tasks compared to their healthy weight peers. Specifically, the RT for inhibition in obese junior high school students was significantly higher [*t*_(50)_ = 3.069, *p* = 0.003, Cohen’s *d* = 0.848] than in healthy weight peers. Additionally, the RT of cognitive flexibility in obese junior high school students was also significantly higher [*t*_(50)_ = 2.600, *p* = 0.013, Cohen’s *d* = 0.706] than in healthy weight peers. However, no significant difference was observed in working memory [*t*_(50)_ = 1.047, *p* = 0.300, Cohen’s *d* = 0.292]. These results indicated that obese junior high school students may have lower EF levels than their healthy weight peers, particularly in the areas of inhibition and cognitive flexibility (see Table 1).

**Table 1:** Results of the Independent-samples *T-*test for developmental characteristics of EF in obese and healthy weight junior high school students (M ± SD).

EF	Obese junior high school students	Healthy weight junior high school students	*t*	*p*	Cohen’s *d*
Inhibition	24.38 ± 13.79	11.57 ± 16.33	3.069	0.003^**^	0.848
Working memory	826.21 ± 223.26	762.57 ± 212.71	1.047	0.300	0.292
Cognitive flexibility	473.63 ± 190.24	367.91 ± 93.08	2.600	0.013^*^	0.706

*p ≤ 0.05, **p ≤ 0.01.

### 3.3 Effects of exercise intervention on EF in obese junior high school students and characteristics of time-course changes

#### 3.3.1 Results of inhibition

The results revealed that the main effect of time on inhibition was significant [*F*_(2,100)_ = 5.181, *p* = 0.01, *η^2^_p_* = 0.094], indicating a trend of change in inhibition over time. The main effect of group on inhibition was significant [*F*_(1,50)_ = 5.203, *p* = 0.027, *η^2^_p_* = 0.094], indicating a significant difference in inhibition between the experimental and control groups. The interaction effect of time * group was also significant [*F*_(2,100)_ = 3.848, *p* = 0.03, *η^2^_p_* = 0.071], indicating a significant difference in the change in inhibition between the experimental and control groups at different time points (see Table 2).

**Table 2:** ANOVA results on the effect of exercise intervention on inhibition in obese junior high school students.

Interaction effect	Type III sum of squares	df	Mean square	*F*	*p*	*η* ^2^_p_
Group	1393.593	1	1393.593	5.203	0.027^*^	0.094
Time	1215.521	2	693.719	5.181	0.01^**^	0.094
Time * group	902.922	2	515.314	3.848	0.03^*^	0.071

*p ≤ 0.05, **p ≤ 0.01.

**Figure 6:**
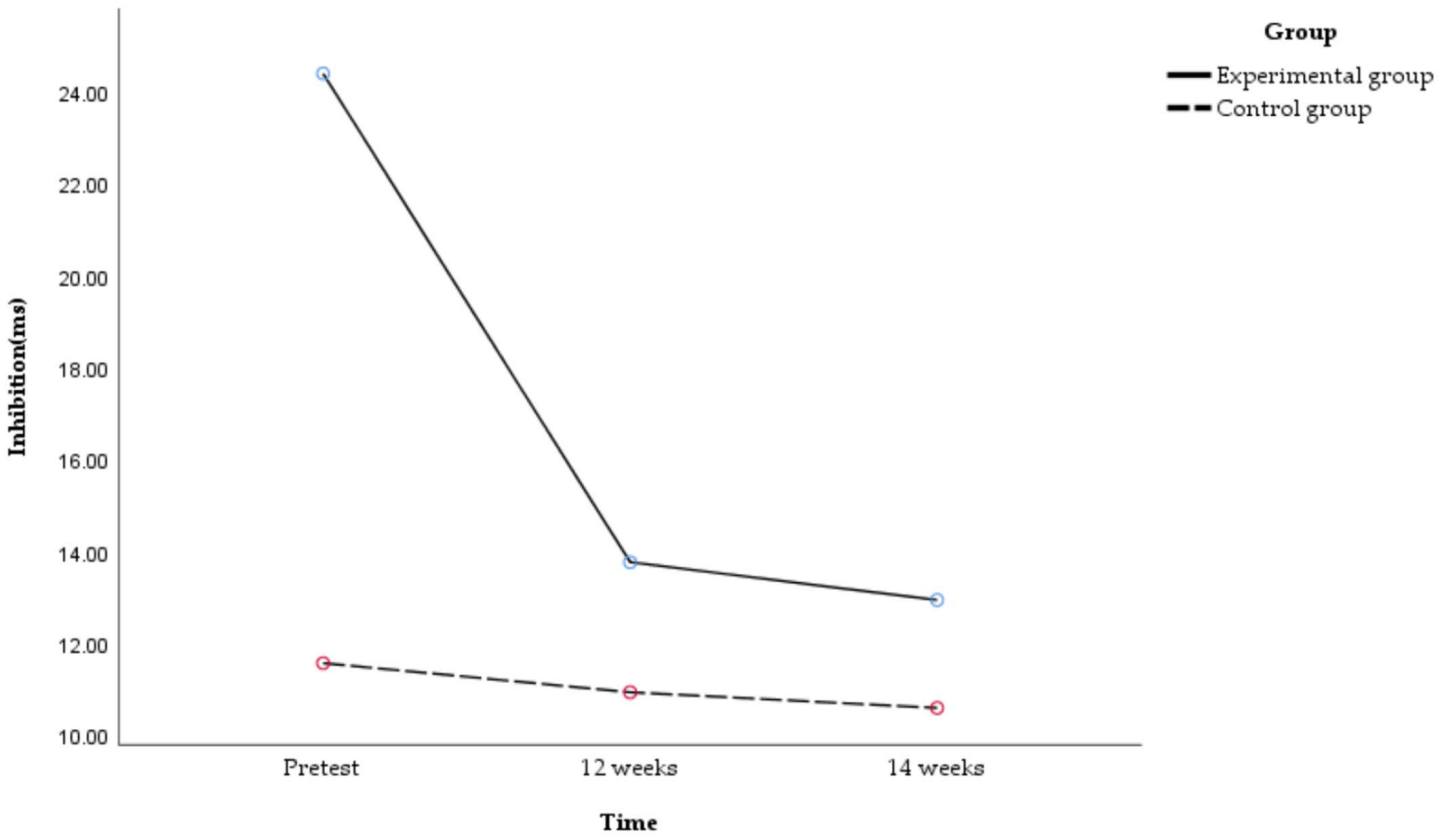
Temporal changes in inhibition in obese and healthy weight junior high school students.

A subsequent analysis of simple effects was conducted, and the following results are shown in Figure 6.

There were significant differences in inhibition at different time points in the experimental group [*F*_(2,49)_ = 10.998, *p* < 0.01, *η^2^_p_* = 0.310]. The inhibition of the experimental group at 12 [*t*_(27)_ = 3.774, *p* < 0.01, Cohen’s *d* = 0.713] and 14 weeks [*t*_(27)_ = 3.613, *p* = 0.003, Cohen’s *d* = 0.683] were significantly better than the pretest, but there was no significant difference between 12 and 14 weeks [*t*_(27)_ = 0.327, *p* = 0.99, Cohen’s *d* = 0.153]. The time-course effect of inhibition showed that 14 and 12 weeks > pretest. That is, the 14-week exercise intervention combining aerobic exercise and resistance training could improve the inhibition of obese junior high school students, and the effect remained stable with the increase in exercise intervention time. Conversely, no significant differences in inhibition were observed across the three time points in the control group [*F*_(2,49)_ = 0.045, *p* = 0.956, *η^2^_p_* = 0.002].

There was a significant difference between obese and healthy weight junior high school students in inhibition at pretest [*F*_(1,50)_ = 9.416, *p* = 0.003, *η^2^_p_* = 0.158], and there was no significant difference in inhibition at 12 [*F*_(1,50)_ = 0.883, *p* = 0.352, *η^2^_p_* = 0.017] or 14 weeks [*F*_(1,50)_ = 0.444, *p* = 0.508, *η^2^_p_* = 0.009]. That is, before the exercise intervention, the inhibition of healthy weight junior high school students was better than that of obese peers. After the exercise intervention, the inhibition of obese junior high school students aligned with that of healthy weight peers across all assessment time points.

A *post hoc* power analysis was conducted using G*Power version 3.1.9.7 (Kang, 2021), with an effect size *f* of 0.276 (partial *η*^2^ as 0.071), α as 0.05, group as 2, number of measurements as 3, and correlation as 0.228. Within our selected total sample size, the power (1-β) was approximately 0.994.

#### 3.3.2 Results of working memory

The results revealed that the main effect of time on working memory was significant [*F*_(2,100)_ = 3.669, *p* = 0.039, *η^2^_p_* = 0.068], indicating a trend of change in working memory over time. Conversely, no significant difference was observed in the main effect of group on working memory [*F*_(1,50)_ = 0.298, *p* = 0.588, *η^2^_p_* = 0.006], and the time * group interaction was also non-significant [*F*_(2,100)_ = 1.181, *p* = 0.304, *η^2^_p_* = 0.023] (see Table 3).

**Table 3:** ANOVA results on the effect of exercise intervention on working memory in obese junior high school students.

Interaction effect	Type III sum of squares	df	Mean square	*F*	*p*	*η* ^2^_p_
Group	17934.972	1	17934.972	0.298	0.588	0.006
Time	128367.279	2	79922.483	3.669	0.039^*^	0.068
Time * group	41336.161	2	25736.221	1.181	0.304	0.023

*p ≤ 0.05, **p ≤ 0.01.

**Figure 7:**
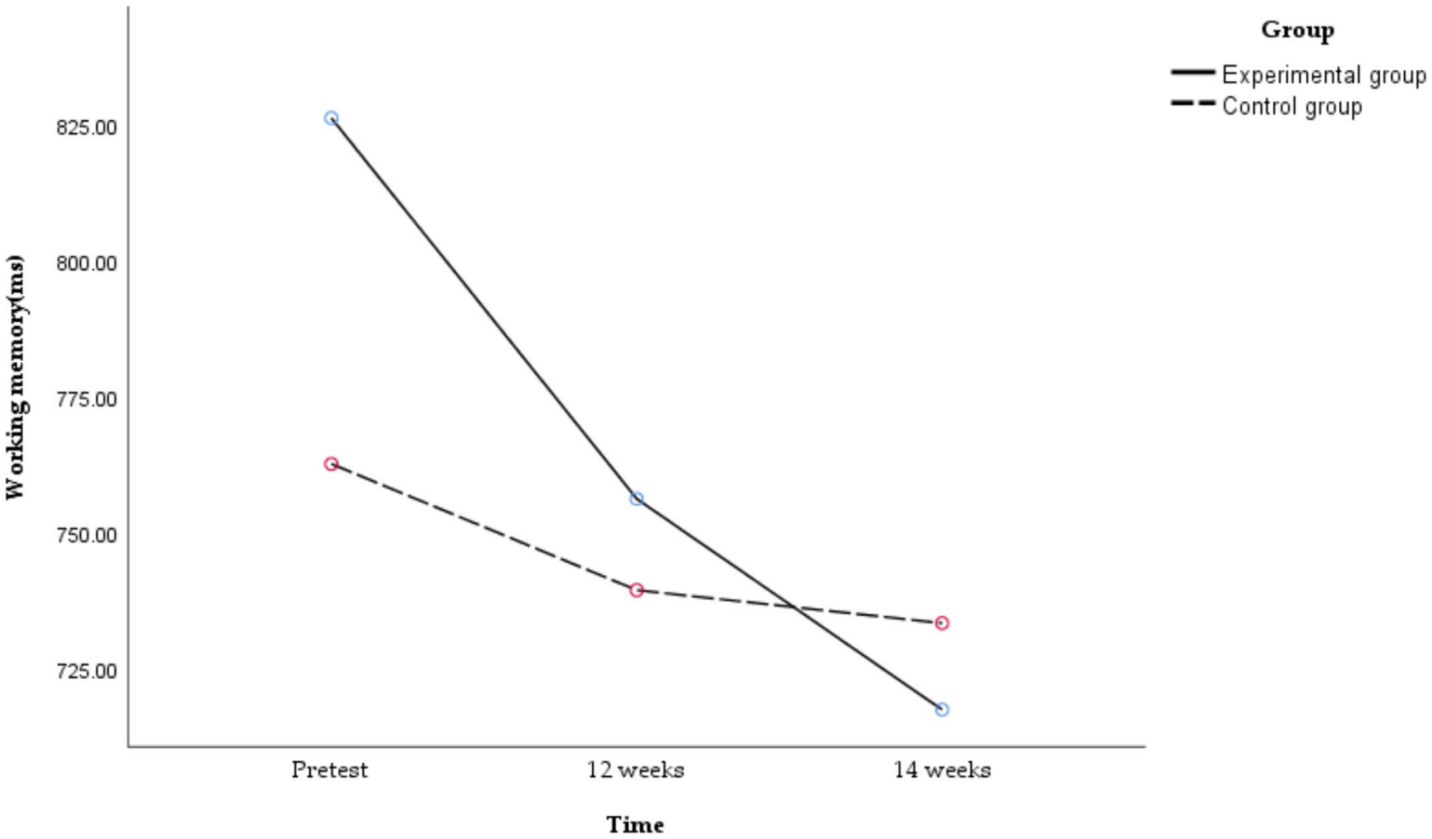
Temporal changes in working memory in obese and healthy weight junior high school students.

A subsequent analysis of simple effects was conducted, and the following results are shown in Figure 7.

There were significant differences in working memory at different time points in the experimental group [*F*_(2,49)_ = 6.403, *p* = 0.003, *η^2^_p_* = 0.207]. The working memory of the experimental group at 14 weeks was significantly better than that at the pretest [*t*_(27)_ = 3.756, *p* = 0.004, Cohen’s *d* = 0.710], but there was no significant difference between 12 weeks and the pretest [*t*_(27)_ = 1.775, *p* = 0.296, Cohen’s *d* = 0.336] and no significant difference between 12 and 14 weeks [*t*_(27)_ = 1.473, *p* = 0.469, Cohen’s *d* = 0.664]. The time-course effect of working memory showed that 14 weeks > 12 weeks, pretest. That is, the 14-week exercise intervention combining aerobic exercise and resistance training could improve the working memory of obese junior high school students, with a longer exercise intervention time correlating with better improvement of working memory. Conversely, no significant differences in working memory were observed across the three time points in the control group [*F*_(2,49)_ = 0.364, *p* = 0.697, *η^2^_p_* = 0.015].

A *post hoc* power analysis was conducted using G*Power version 3.1.9.7 (Kang, 2021), with an effect size f of 0.153 (partial *η*^2^ as 0.023), *α* as 0.05, group as 2, number of measurements as 3, and correlation as 0.619. Within our selected total sample size, the power (1-β) was approximately 0.661.

#### 3.3.3 Results of cognitive flexibility

The results revealed that the main effect of time was significant [*F*_(2,100)_ = 12.242, *p* < 0.01, *η^2^_p_* = 0.197], indicating a trend of change in cognitive flexibility over time. The interaction effect of time * group was also significant [*F*_(2,100)_ = 3.913, *p* = 0.023, *η^2^_p_* = 0.073], indicating a significant difference in the change in cognitive flexibility between the experimental and control groups at different time points. Conversely, the group main effect was not significant [*F*_(1,50)_ = 2.164, *p* = 0.148, *η^2^_p_* = 0.041] (see Table 4).

**Table 4:** ANOVA results on the effect of exercise intervention on cognitive flexibility in obese junior high school students.

Interaction effect	Type III sum of squares	df	Mean square	*F*	*p*	*η* ^2^_p_
Group	101169.019	1	101169.019	2.164	0.148	0.041
Time	209111.331	2	104555.666	12.242	<0.01**	0.197
Time * group	66835.015	2	33417.507	3.913	0.023^*^	0.073

*p ≤ 0.05, **p ≤ 0.01.

**Figure 8:**
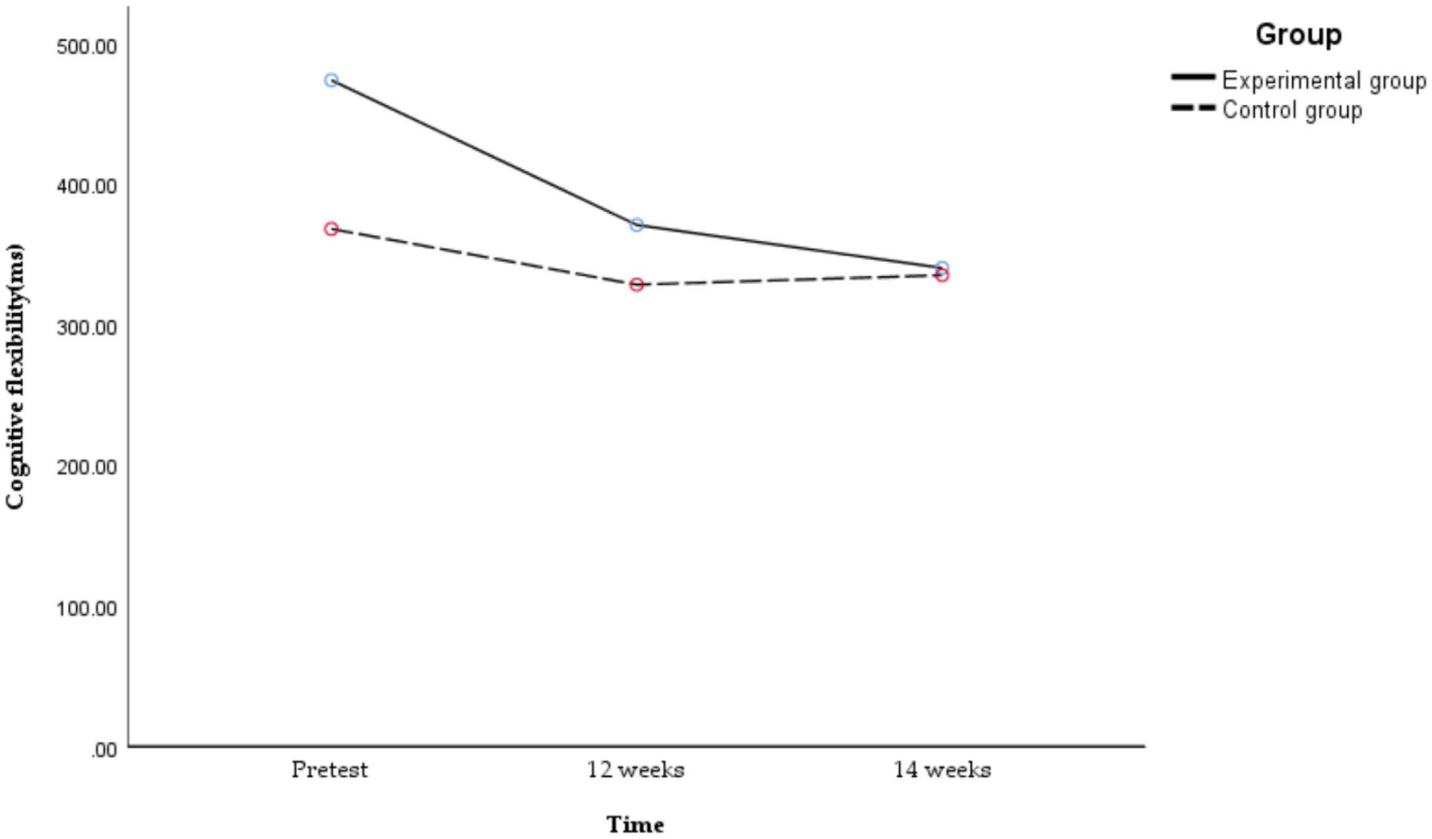
Temporal changes in cognitive flexibility in obese and healthy weight junior high school students.

A subsequent analysis of simple effects was conducted, and the following results are shown in Figure 8.

There were significant differences in cognitive flexibility at different time points in the experimental group [*F*_(2,49)_ = 14.160, *p* < 0.01, *η^2^_p_* = 0.366]. The cognitive flexibility of the experimental group at 12 [*t*_(27)_ = 4.217, *p* = 0.001, Cohen’s *d* = 0.797] and 14 weeks were significantly better than the pretest [*t*_(27)_ = 4.714, *p* < 0.01, Cohen’s *d* = 0.890], but there was no significant difference between 12 and 14 weeks [*t*_(27)_ = 1.900, *p* = 0.416, Cohen’s *d* = 0.359]. The time-course effect of cognitive flexibility showed that 14 and 12 weeks > pretest. That is, the 14-week exercise intervention combining aerobic exercise and resistance training could improve the cognitive flexibility of obese junior high school students, and the effect remained stable with the increase in exercise intervention time. Conversely, no significant differences in cognitive flexibility were observed across the three time points in the control group [*F*_(2,49)_ = 0.985, *p* = 0.381, *η^2^_p_* = 0.039].

There was a significant difference between obese and healthy weight junior high school students in cognitive flexibility at pretest [*F*_(2,100)_ = 6.14, *p* = 0.017, *η^2^_p_* = 0.109], and there was no significant difference in cognitive flexibility at 12 [*F*_(2,100)_ = 1.18, *p* = 0.282, *η^2^_p_* = 0.023] or 14 weeks [*F*_(2,100)_ = 0.02, *p* = 0.898, *η^2^_p_* = 0.000]. That is, before the exercise intervention, the cognitive flexibility of healthy weight junior high school students was better than that of obese peers. After the exercise intervention, the cognitive flexibility of obese junior high school students aligned with that of healthy weight peers across all assessment time points.

A *post hoc* power analysis was conducted using G*Power version 3.1.9.7 (Kang, 2021), with an Effect size *f* of 0.281 (partial *η*^2^ as 0.073), *α* as 0.05, group as 2, number of measurements as 3, and correlation as 0.581. Within our selected total sample size, the power (1-β) was approximately 0.995.

### 3.4 Effects of exercise intervention on BMI and BFR of obese junior high school students

#### 3.4.1 Comparison of BMI and BFR between obese junior high school students and healthy weight junior high school students

To investigate the difference of BMI and BFR between obese and healthy weight junior high school students, the Independent-samples *T-*test was conducted for BMI and BFR of the pretests. The results revealed that the BMI [*t*_(50)_ = 16.527, *p* < 0.01, Cohen’s *d* = 4.606] and BFR [*t*_(50)_ = 13.537, *p* < 0.01, Cohen’s *d* = 3.781] of obese junior high school students were higher than those of healthy peers, and the difference was significant. That is, the BMI and BFR of obese junior high school students were significantly higher than those of healthy weigh peers (see Table 5).

**Table 5:** Results of the independent-samples *T-*test of BMI and BFR in obese and healthy weight junior high school students (M ± SD).

Index	Obese junior high school students	Healthy weight junior high school students	*t*	*p*	Cohen’s *d*
BMI	28.75 ± 2.42	17.85 ± 2.30	16.527	<0.01**	4.606
BFR	39.50 ± 6.22	17.07 ± 5.63	13.537	<0.01**	3.781

*p ≤ 0.05, **p ≤ 0.01.

#### 3.4.2 Effects of exercise intervention on BMI of obese junior high school students

The repeated-measures ANOVA was used with the following results to analyze the effects of the exercise intervention on BMI in obese junior high school students.

The results revealed that the main effect of time on BMI was nearly significant [*F*_(1,50)_ = 3.998, *p* = 0.051, *η^2^_p_* = 0.074], indicating a trend of change in BMI over time. The main effect of group on BMI was significant [*F*_(1,50)_ = 216.342, *p* < 0.01, *η^2^_p_* = 0.812], indicating a significant difference in BMI between the experimental and control groups. The interaction effect of time * group was also significant [*F*_(1,50)_ = 23.988, *p* < 0.01, *η^2^_p_* = 0.324], indicating a significant difference in the change in BMI between the experimental and control groups at pretest and 14 weeks (see Table 6).

**Table 6:** ANOVA results on the effect of exercise intervention on BMI in obese junior high school students.

Interaction effect	Type III sum of squares	df	Mean square	*F*	*p*	*η* ^2^_p_
Group	2399.886	1	2399.886	216.342	<0.01**	0.812
Time	6.844	1	6.844	3.998	0.051	0.074
Time * group	41.060	1	41.060	23.988	<0.01**	0.324

*p ≤ 0.05, **p ≤ 0.01.

**Figure 9:**
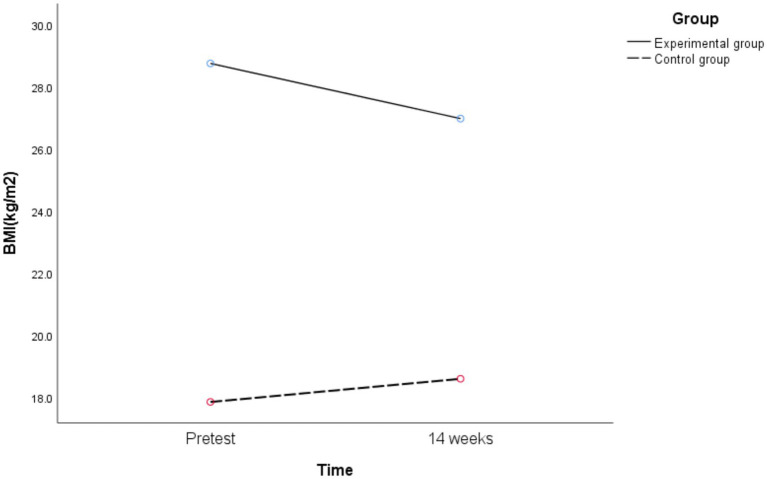
Changes of BMI in different groups before and after exercise intervention.

Further simple effects analysis was performed, and the following results are shown in Figure 9.

The 14 weeks BMI of obese junior high school students in the experimental group was significantly different from the pretest [*t*_(27)_ = 4.312, *p* < 0.01, Cohen’s *d* = 0.815], while the 14 weeks BMI of healthy weight junior high school students in the control group was significantly different from the pretest [*t*_(23)_ = −2.670, *p* = 0.014, Cohen’s *d* = 0.545]. That is, the 14-week of exercise intervention combining aerobic exercise and resistance training can improve the BMI of obese junior high school students, while the BMI of healthy weight junior high school students has an upward trend.

There was a significant difference between the experimental group and the control group in BMI at pretest [*F*_(1,50)_ = 273.13, *p* < 0.01, *η^2^_p_* = 0.845], and there was also a significant difference in BMI at 14 weeks [*F*_(1,50)_ = 126.14, *p* < 0.01, *η^2^_p_* = 0.716]. That is, before exercise intervention, the BMI of healthy weight junior high school students in the control group was better than that of obese junior high school students in the experimental group. After exercise intervention, the BMI of healthy weight junior high school students in the control group was still better than that of obese junior high school students in the experimental group.

#### 3.4.3 Effects of exercise intervention on BFR of obese junior high school students

The repeated-measures ANOVA was used with the following results to analyze the effects of the exercise intervention on BFR in obese junior high school students.

The results revealed that the main effect of time on BFR was significant [*F*_(1,50)_ = 17.617, *p* = 0.046, *η^2^_p_* = 0.077], indicating a trend of change in BFR over time. The main effect of group on BFR was significant [*F*_(1,50)_ = 138.144, *p* < 0.01, *η^2^_p_* = 0.734], indicating a significant difference in BFR between the experimental and control groups. The interaction effect of time * group was also significant [*F*_(1,50)_ = 438.140, *p* < 0.01, *η^2^_p_* = 0.675], indicating a significant difference in BFR between the experimental and control groups at pretest and 14 weeks (see Table 7).

**Table 7:** ANOVA results on the effect of exercise intervention on BFR in obese junior high school students.

Interaction effect	Type III sum of squares	df	Mean square	*F*	*p*	*η* ^2^_p_
Group	8667.447	1	8667.447	138.144	<0.01**	0.734
Time	17.617	1	17.617	4.178	0.046*	0.077
Time * group	438.140	1	438.140	103.912	<0.01**	0.675

*p ≤ 0.05, **p ≤ 0.01.

**Figure 10:**
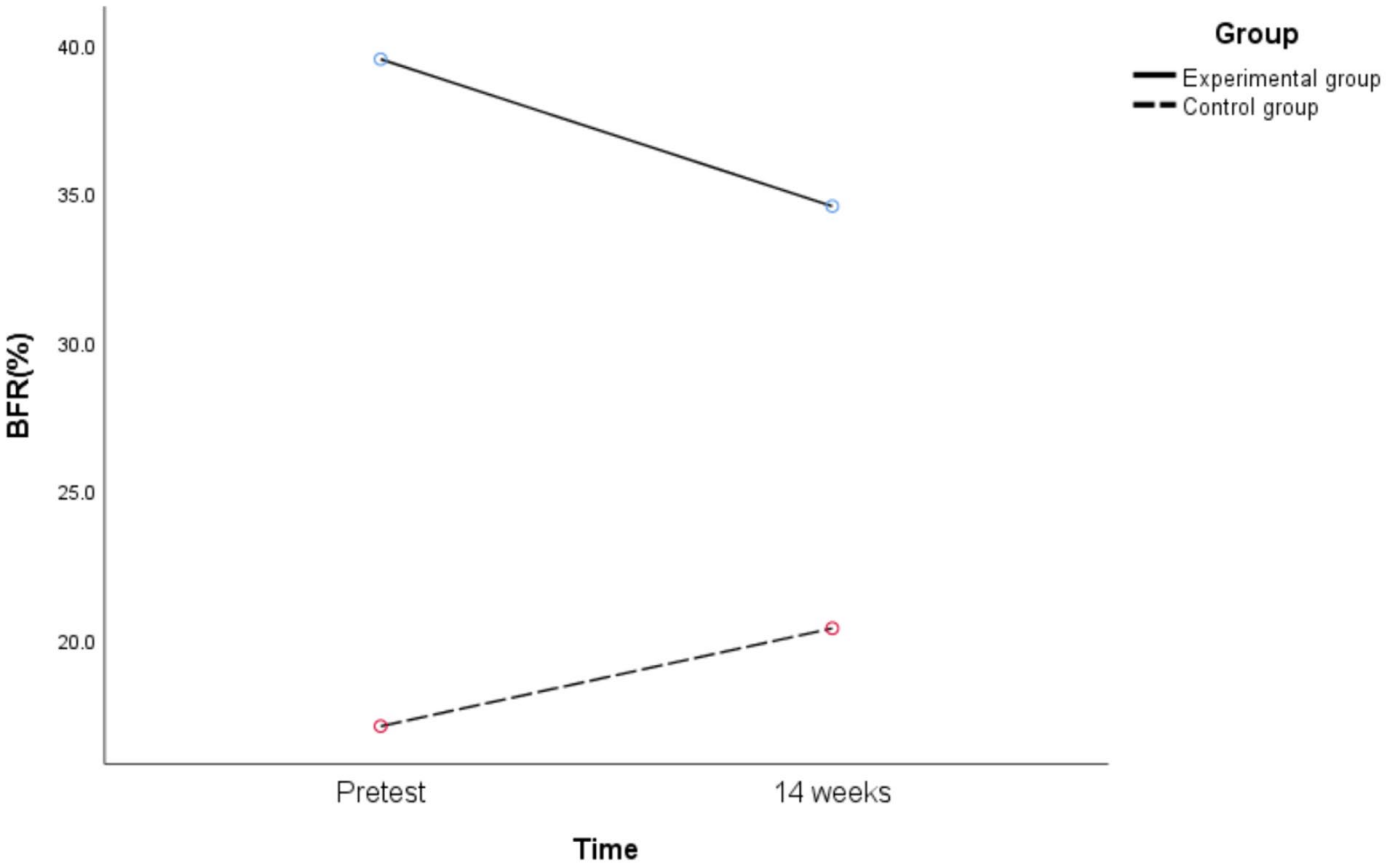
Changes of BFR in different groups before and after exercise intervention.

Further simple effects analysis was performed, and the following results are shown in Figure 10.

The 14 weeks BFR of obese junior high school students in the experimental group was significantly different from the pretest [*t*_(27)_ = 8.910, *p* < 0.01, Cohen’s *d* = 1.685], while the 14 weeks BFR of healthy weight junior high school students in the control group was significantly different from the pretest [*t*_(23)_ = −5.625, *p* < 0.01, Cohen’s *d* = 1.146]. That is, the 14-week exercise intervention combining aerobic exercise and resistance training can improve the BFR of obese junior high school students, while the BFR of healthy weight junior high school students has an upward trend.

There was a significant difference between the experimental group and the control group in BFR at pretest [*F*_(1,50)_ = 183.257, *p* < 0.01, *η^2^_p_* = 0.786], and there was also a significant difference in BFR at 14 weeks [*F*_(1,50)_ = 82.72, *p* < 0.01, *η^2^_p_* = 0.623]. That is, before exercise intervention, the BFR of healthy weight junior high school students in the control group was better than that of obese junior high school students in the experimental group. After exercise intervention, the BFR of healthy weight junior high school students in the control group was still better than that of obese junior high school students in the experimental group.

## 4 Discussion

### 4.1 Analysis of the developmental characteristics of EF in obese junior high school students

This study supported the hypothesis that obese adolescents exhibit impaired EF compared to their healthy weight peers, particularly in inhibition and cognitive flexibility. These findings aligned with previous research demonstrating similar findings in obese adolescents. Gentier et al. (2013) found EF impairment and motor perception dysfunction in obese children compared to healthy weight children, and Martí-Nicolovius (2022) found deficits in cognitive function in obese children and adolescents, further highlighting the pervasive impact of obesity on cognitive abilities.

The physiological mechanisms behind the poor development of EF in obese junior high school students may be that obese people have a high fat content, which leads to a significant reduction in cerebral blood flow and speed, insufficient blood supply to the brain, and a decrease in blood oxygen saturation, which affects their brain metabolic processes (Ebbeling et al., 2002; Guo and Chen, 2015). In terms of brain mechanisms, obese people have reduced volume and functional connectivity in brain areas related to EF (Willette and Kapogiannis, 2015), resulting in damage to the wiring system connecting the information processing areas of the brain, which in turn hinders the transmission of signals, leading to slow thinking and slow response, which negatively affects the development of EF.

The implications of our findings were profound, indicating that the compromised EF in obese adolescents could have broader consequences for their overall health and well-being. Given the pivotal role of EF in academic achievement and social adaptation, addressing this issue was not only crucial for the students’ immediate cognitive development but also for their long-term success and mental health. This study thus called for the development and implementation of targeted interventions aimed at enhancing EF in obese adolescents.

### 4.2 Time-course effects of exercise intervention combining aerobic exercise with resistance training on EF in obese junior high school students

This study supported the hypothesis that exercise intervention combining aerobic exercise with resistance training can significantly enhances EF in obese adolescents. These findings aligned with previous research indicating the cognitive benefits of both aerobic and resistance training for obese adolescents (Guo et al., 2016). However, this study stood out by adopting an innovative approach that combines these two exercise modalities, focusing on three key pathways—action characteristics, exercise intensity, and exercise scenarios—to optimize EF improvement. The selection of complex, novel, and targeted actions, adjusted dynamically to the external environment, along with moderate-intensity exercise and diverse scenarios, constituted the unique aspect of our intervention. This multifaceted approach effectively improved EF in obese adolescents.

Beyond the three key pathways, the physiological mechanisms underlying our exercise intervention’ s efficacy in improving EF warrant attention. Long-term aerobic exercise combined with resistance training intervention successfully reduced fat content, enhanced blood circulation, and improved blood oxygen saturation in obese junior high school students. These physiological changes activated brain regions and functional connectivity associated with EF, as well as enhanced hippocampal insulin signaling and neuroplasticity (Chen et al., 2011a,b; Guo et al., 2016; Park et al., 2019). These findings contributed to the understanding of how combined exercise interventions can specifically target and improve EF in obese adolescents.

Utilizing a multi-time-point measurement design, our study uniquely explored the time-course effects of the exercise intervention on the three sub-functions of EF: inhibition, working memory, and cognitive flexibility. Our results indicated that different intervention cycles had selective positive effects on these sub-functions. Notably, inhibition and cognitive flexibility significantly improved at 12 weeks, while working memory significantly improved at 14 weeks. These findings build upon Yin et al.’s (2014) study, suggested that longer intervention durations may yield better EF outcomes. This study underscored the importance of identifying optimal time points for significant EF changes.

Throughout the exercise intervention, participants engaged in continuous inhibition of dominant responses and conscious muscle control during resistance training, which likely contributed to the significant improvements in inhibition. Cognitive flexibility was enhanced through the need for constant movement transitions in response to changing exercise commands and rules. Working memory improvement was attributed to the participants’ requirement to memorize movements and game rules, and responded quickly in dynamic situations. Our focus on improving inhibition and cognitive flexibility, areas typically weaker in obese students compared to their healthy weight peers, may explained the earlier observed changes in these sub-functions. This targeted approach to exercise intervention design represents a novel strategy for optimizing EF development in obese adolescents.

### 4.3 The strengths and the limitations of this study

This study’s novel contribution lied in the specific focus on the time-course effects of the exercise intervention. This provided valuable insights into the optimal timing and duration of exercise interventions for EF development in obese adolescents. Additionally, the combination of aerobic exercise and resistance training, along with the multifaceted approach incorporating action complexity and diverse exercise scenarios, offered a unique perspective on the potential mechanisms underlying the observed improvements. In addition, the health implications of this study were significant. Improved EF can enhance academic performance, social interactions, and overall cognitive functioning, ultimately improving the quality of life for obese adolescents. This study provided evidence supporting the potential of exercise as a non-pharmacological intervention for improving cognitive function and overall health in obese adolescents.

Despite its strengths, this study had limitations. The relatively shorted duration of the intervention and limited number of time points for EF measurements warrant further research exploring the long-term effects of exercise on the development of EF in obese adolescents as well as the late effects. Additionally, increasing the sample size and incorporating brain imaging techniques could provide valuable insights into the neural mechanisms underlying the observed improvements. Future research could also explored the impact of exercise on other cognitive functions, such as attention and memory, in obese adolescents. Additionally, investigating the effectiveness of targeted exercise interventions for different sports could provide valuable insights into the optimal exercise prescription for improving EF in obese adolescents.

## 5 Conclusion

Obese adolescents had impaired EF, as evidenced by low levels of the inhibition and cognitive flexibility compared to healthy weight adolescents. The exercise intervention combining aerobic exercise and resistance training had a positive effect on EF of obese adolescents. The time-course effects of the intervention on improvements in inhibition, working memory, and cognitive flexibility varied with intervention duration in obese adolescents, with significant changes in inhibition and cognitive flexibility observed at 12 weeks and significant changes in working memory at 14 weeks.

## Data Availability

The original contributions presented in the study are included in the article/supplementary material, further inquiries can be directed to the corresponding authors.
